# Ultra-thin (<2 µm) silicon carbide free-standing membranes as beam position monitors for soft and tender X-ray beamlines

**DOI:** 10.1107/S1600577525007362

**Published:** 2025-10-27

**Authors:** E. Medina, G. Trovato, L. Calcagno, S. Kalbfleisch, M. Birri, G. Milluzzo, F. Romano, E. Sangregorio, S. Moscato, F. M. Milian, S. Giordanengo, A. Vignati, M. Camarda

**Affiliations:** a Università degli Studi di Torino via P. Giuria 1 10125Torino Italy; b INFN, Sezione di Torino, 10125Torino, Italy; c STLab srl, Via Anapo 53, 95126Catania, Italy; d Institut de Física d’Altes Energies (IFAE) The Barcelona Institute of Science and Technology Edifici CN, UAB Campus 08193Bellaterra(Barcelona) Spain; e Dipartimento di Fisica e Astronomia Ettore Majorana Università degli studi di Catania Via Santa Sofia 64 95123Catania Italy; f Istituto per la Microelettronica e Microsistemi CNR-IMM Sezione di Catania, Strada VIII Zona Industriale 5 95121Catania Italy; g Istituto Nazionale di Fisica Nucleare – INFN Sezione di Catania, Via S. Sofia 64 95123Catania Italy; h MAX IV Laboratory Lund University PO Box 118 S-221 00Lund Sweden; i Paul Scherrer Institute Forschungsstrasse 111 5232Villigen PSI Switzerland; j SenSiC GmbH, DeliveryLAB, Villigen, Switzerland; SESAME, Jordan

**Keywords:** beam position monitors, silicon carbide, radiation detector, beamline instrumentation, X-rays, detector simulations

## Abstract

Conventional beam intensity monitor technologies, such as ‘gold-meshes’ and ‘diamond conductive thin films’, currently applied to tender and soft X-ray beams, encounter numerous application challenges, including diffraction effects, low signal strength, non-uniform transparency, and lack of position information. This study explores the potential of very thin (<2 µm) silicon carbide free-standing membranes, as in-line, minimally interfering beam intensity and position monitors, with high-lateral resolution, for soft and tender X-ray beamlines. Initial experimental assessments were conducted at the NanoMAX beamline at MAX IV to analyze the performance of such very thin devices in monitoring tightly focused (<1 µm FWHM) beams. The tests revealed that employing four-quadrant sensor layouts on such thin sensors resulted in significant charge collection losses in the regions between the quadrants and, under high electric field conditions, in charge multiplication effects (avalanche effects). Through Sentaurus TCAD theoretical simulations, the limitations of the four-quadrant design for such applications and the potential of an alternative technology (resistive X-ray beam postion monitor) were clarified.

## Abstract 2

The use of ultra-thin (<2 µm) silicon carbide membranes as in-line beam intensity and position monitors for soft and tender X-ray beamlines is investigated. Experimental results and theoretical simulations highlight the limitations of four-quadrant sensor designs, and alternative solutions for high-resolution and minimally interfering beam monitoring are proposed.

## 1. Introduction

Synchrotron beamlines are highly versatile tools for a wide range of scientific measurements using X-rays, offering invaluable insights into the structural, electronic, and magnetic properties of materials. These facilities excel due to their ability to provide continuously tunable photon energies with high resolution, a broad energy range spanning from infrared photons to hard X-rays, exceptional coherence, and extremely high brilliance. This combination of features enables a wide array of applications, including X-ray absorption spectroscopy (XAS), diffraction, imaging, scattering, and tomography. By probing critical absorption edges of elements across the periodic table, synchrotron radiation facilitates studies of atomic structure, chemical bonding, electronic states, and dynamic processes, making it an essential tool for advancing materials science, biology, environmental and industrial research.

Many synchrotron facilities operate dedicated beamlines for X-ray absorption experiments in the range from 10–12 keV down to a few hundred eV. To ensure accurate and quantitative analysis of the X-ray absorption data, it is crucial to precisely monitor the photon flux, and, ideally, the position of the beam impinging on the sample. In particular, the flux can significantly fluctuate with factors like the storage ring current and incoming light polarization whereas the beam position can change due to vibrations or drifts of the optical elements, *e.g.* monochromators. As a result, these beamlines are often equipped with specialized beam intensity and beam position monitors, generally referred to as XBIMs and XBPMs, respectively, as the final optical components before the sample or along the beam path. A successful XBIM/XBPM should exhibit linearity of the signal in relation to the transmitted beam, offer a substantial monitor signal akin to the sample signal, and simultaneously maintain a high beam transmission ratio without distorting the beam shape.

Current XBIM technologies for soft and tender X-ray beams predominantly rely on ‘gold meshes’ and ‘diamond conductive thin films’ (Kummer *et al.*, 2013 ▸). However, both suffer from significant drawbacks, including very low quantum efficiencies that result in weak signals and poor monitoring accuracy. Mesh-based XBIM technologies also introduce diffraction patterns, which become more pronounced as beam sizes decrease, leading to signal distortions and a loss of linearity. While these sensors are effective as intensity monitors, they are not designed to provide the increasingly necessary spatial tracking of the transmitted beam. Such tracking is crucial for applications like active feedback loops to counteract beam position drifts, ensuring stable intensity on the sample and consistent results, particularly for non-uniform samples.

At present, no XBPM technology is available for soft and tender X-rays, as no existing sensor offers adequate transparency in these energy ranges. These limitations have restricted the suitability of current sensors for both XBIM and XBPM applications, underscoring the need for innovative approaches to improve signal fidelity and provide spatial information. In recent years, silicon carbide (SiC) has emerged as an ideal semiconductor material for sensors operating in harsh environments, owing to its exceptional properties (Tudisco *et al.*, 2018 ▸; Medina *et al.*, 2023 ▸; Romano *et al.*, 2023 ▸; Milluzzo *et al.*, 2024 ▸). In more detail, a particular type of SiC sensor, based on free-standing membranes, has emerged as a promising alternative to diamond CVD membranes for XBPM applications in hard X-rays (Desjardins *et al.*, 2014 ▸; Griesmayer *et al.*, 2019 ▸). SiC membranes are commercially available with thicknesses of 2 µm, 10 µm, and 20 µm (Nida *et al.*, 2019 ▸; SenSiC, 2021 ▸; Trovato *et al.*, 2025 ▸). Due to the shorter attenuation lengths of SiC compared with diamond (see https://cividec.at/detectors-B9.html), significantly thinner SiC membranes – approximately ten times thinner – are needed to achieve comparable transparency (Henke *et al.*, 1993 ▸). For example, the thinnest available diamond XBPM, at 20 µm, as well as the 2 µm SiC ones, cause excessive absorption in the soft and tender X-ray range, making it unsuitable.

This study presents an experimental and theoretical analysis of ultra-thin, free-standing SiC membranes (<2 µm) designed to outperform 20 µm diamond XBPMs in terms of transparencies to be used as real-time, inline, monitors for soft and tender X-ray beams. In this paper, these membranes have been evaluated as an innovative solution for both beam position and intensity monitoring. Additionally, an advanced technology computer aided design (TCAD) device simulator, *Sentaurus Synopsys* (Singh & Pandey, 2023 ▸), was used to support and interpret the experimental findings and to study the resistive-XBPM as an alternative technology.

## 2. Materials and methods

**Figure 1:**
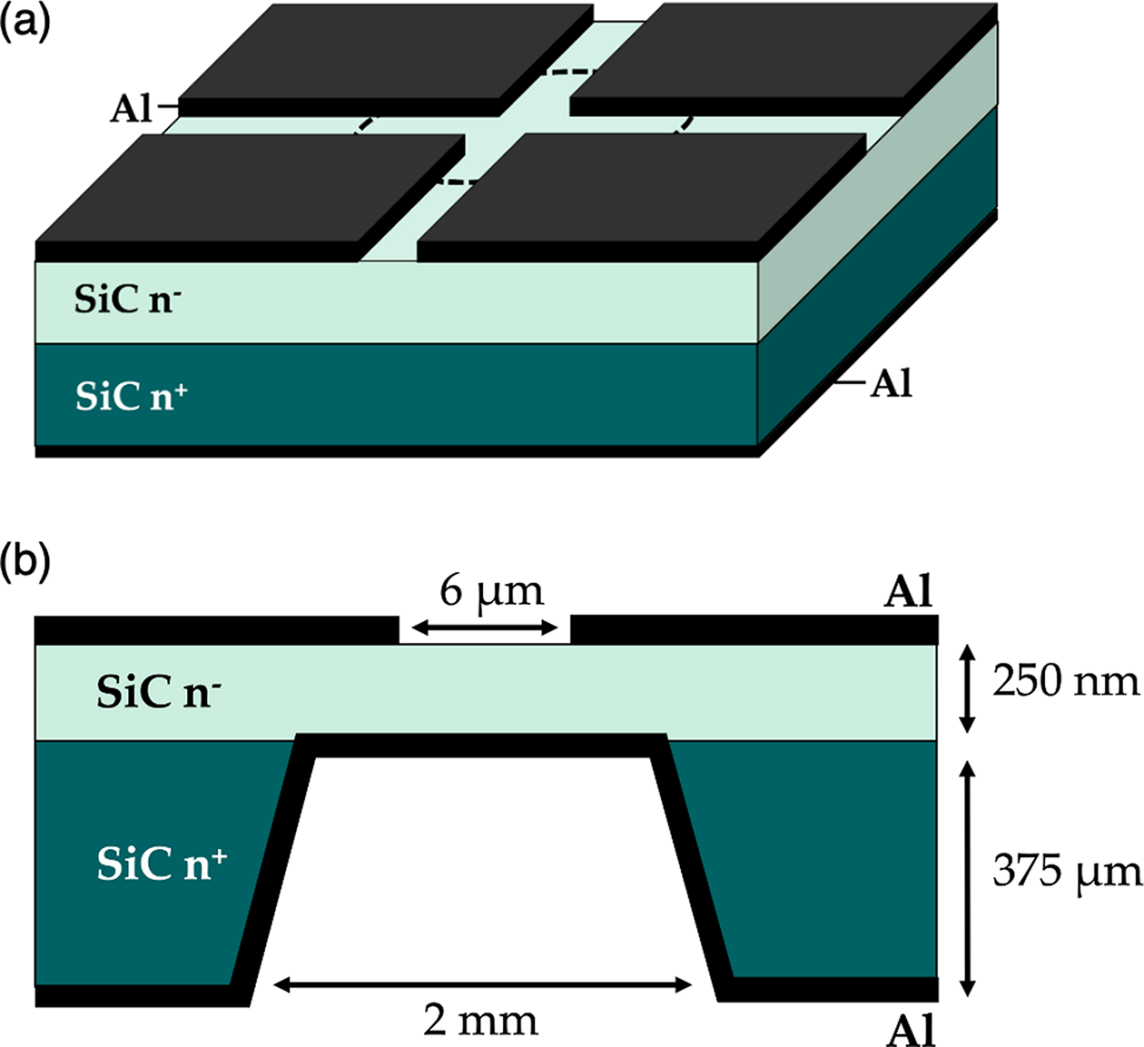
(*a*) The 3D structure (not to scale) of the SiC XBPM device, featuring an n-type layer and a thick n^+^ substrate. (*b*) A 2D cross-section of the device (not to scale), showing the distance between the readout pixels (6 µm), the thickness of the n-type epitaxial layer (250 nm), and the n^+^ substrate (375 µm). The central region around the four pads (circular area of approximately 2 mm in diameter) is thinned using electrochemical dopant-selective etching.

A standard ‘four-quadrant’ XBPM sensor consists of four independent diodes, in the specific case considered 4H-SiC Schottky diodes, characterized by a 375 µm-thick n-type (doping concentration: ∼10^18^ cm^−3^) substrate, and a nominally 250 nm thick n-type epitaxial layer (doping concentration: ∼5 × 10^13^ cm^−3^). The peculiarity of the sensors presented here is that the n^+^ layer is locally removed by an electrochemical doping-selective etching process, creating a thinned-down area in a selected region of the sensor, referred to as free-standing membrane regions (Nida *et al.*, 2019 ▸). The sensor used in this work has four readout pixels on its surface, each spaced 6 µm apart, to monitor the position of the beam as a function of the current measured on each of the pixels and a free-standing central membrane of 2 mm diameter (see Fig. 1 ▸).

**Figure 2:**
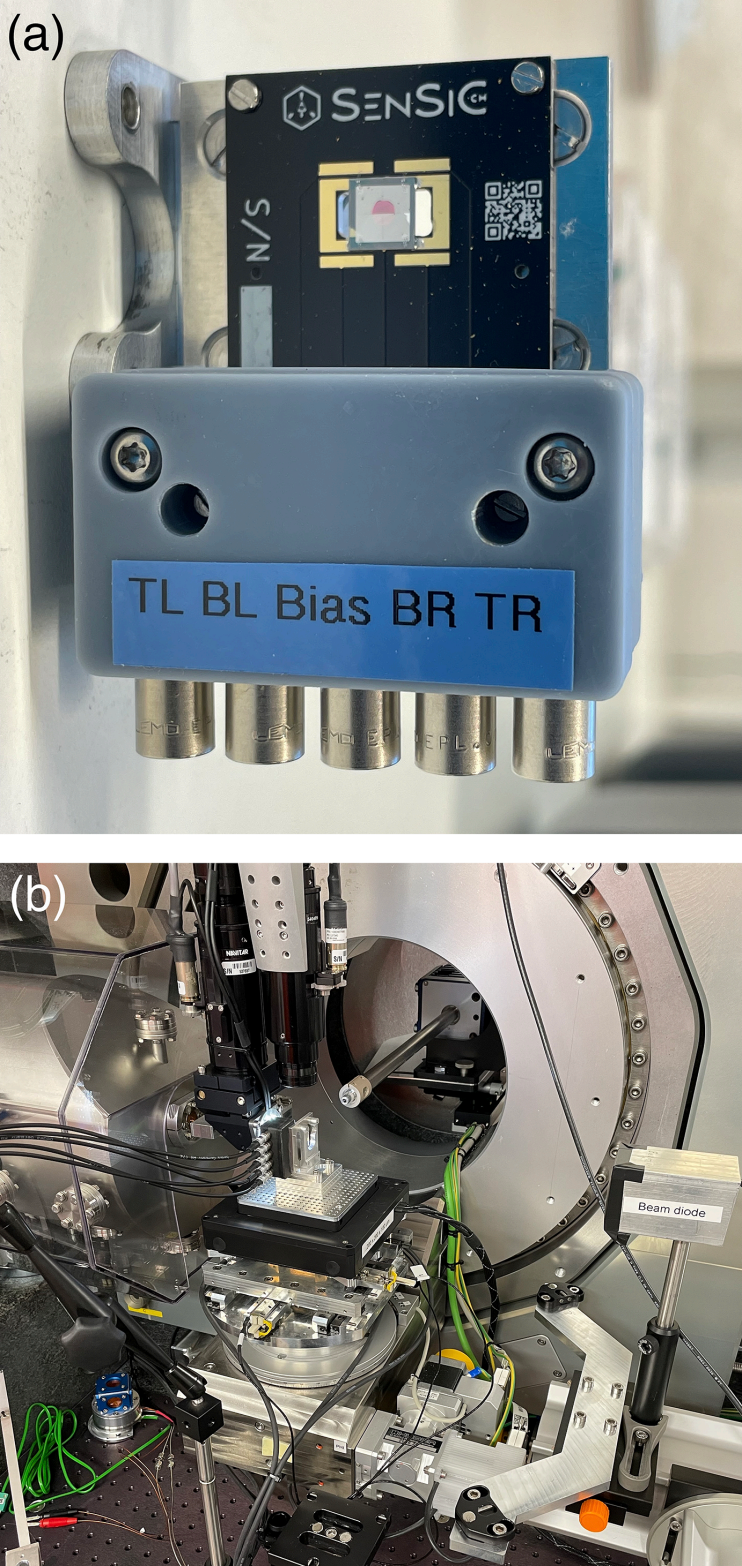
Photographs of the experimental setup at NanoMAX beamline. The SiC sensor (*a*) is mounted in the hard X-ray monochromatic nanoprobe experimental station (*b*).

The first experimental characterization of the lateral resolution of these extremely thin SiC sensors was carried out at the diffraction endstation of the NanoMAX beamline at MAX IV, specifically designed for high-resolution X-ray imaging (Carbone *et al.*, 2022 ▸; Johansson *et al.*, 2021 ▸). The reason for the choice of this beamline is the possibility of delivering highly focused, sub-micrometre spots. This characteristic is ideal to achieve extremely detailed characterizations of the lateral signal response of these ultra-thin SiC sensors. The photon energy was set to 8 keV, and the X-ray beam was focused to a spot size of 110 nm × 110 nm (FWHM, horizontal × vertical) using a pair of Kirkpatrick–Baez (KB) mirrors (Björling *et al.*, 2020 ▸). The SiC XBPM sensor was precisely positioned at the focal plane using the beamline’s optical on-axis microscope. The signal generated by the four electrodes of the SiC sensor was recorded using a low-noise electrometer (Matilla *et al.*, 2015 ▸), configured with a sensitivity range of 1 µA for each channel and a low-pass filter cutoff at 3200 Hz to minimize electronic noise. A conventional DC power supply was used to apply the bias voltage across the device. During each acquisition, the signal was integrated over a duration of 0.1 s. To reduce background contributions, all measurements were performed with the ceiling lights turned off and the microscope illumination disabled. Furthermore, for all presented results, the dark current, measured at a bias voltage of 5 V, was subtracted to isolate the photon-induced response of the device. The experimental setup is illustrated in Fig. 2 ▸.

Device simulations were performed using TCAD *Sentaurus Synopsys*, a new generation device simulator for designing and optimizing current and future semiconductor devices, to study the charge collection efficiency (CCE) as a function of position of the beam on the device. For such simulations, a simplified, two-dimensional sensor geometry was implemented. A 4H-SiC layer, doped with a low nitro­gen concentration of 5 × 10^13^ cm^−3^, was included between a uniform cathode on one side and two symmetrical anodes at the edges of the opposite side. The simulated sensor had a width of 10 µm, with thicknesses varying from 100 nm to 6 µm, to account for how they affect measurements, and the distance between anodes, ranging from 100 nm to 9 µm, to evaluate the impact of inter-pad spacing. The three electrodes are defined as Schottky contacts, with a work function equal to 4.8 eV. A highly collimated 8 keV X-ray beam, with a full width at half-maximum (FWHM) of 100 nm × 100 nm, and a photon flux of 1 × 10^12^ photons s^−1^, was used in the simulation. A beam scan is performed by moving the center of the Gaussian beam horizontally across the XBPM, from one anode to the other. The response of the sensor to changes in sensor thickness and bias voltage was investigated. The results of studies carried out using simulations are described in the next section.

## 3. Results and discussion

**Figure 3:**
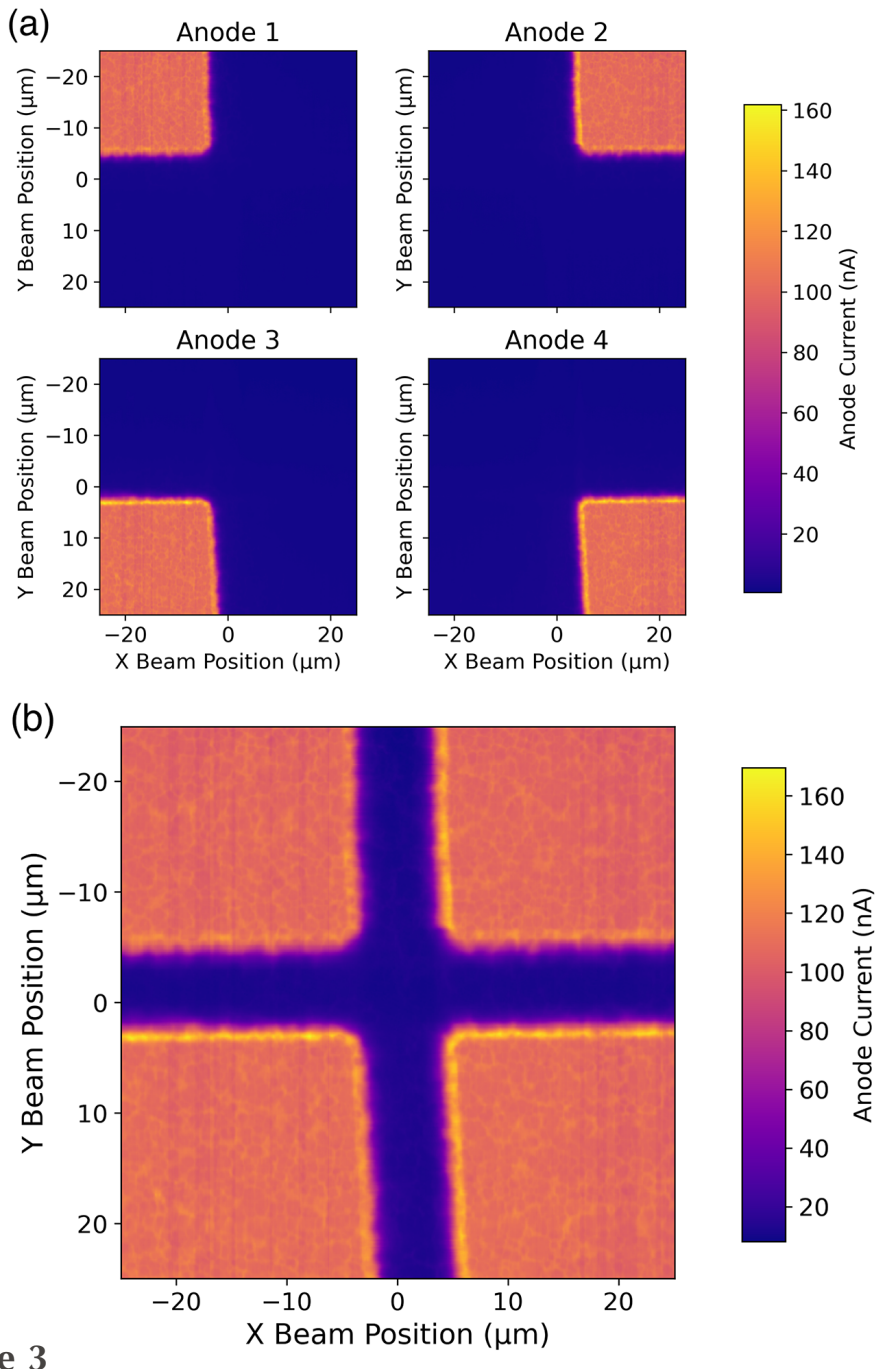
(*a*) Current measured on the individual sensor electrodes (Anode 1, Anode 2, Anode 3, Anode 4) when scanning an 8 keV X-ray beam on its surface. (*b*) Total current measured on the sensor computed as the sum of the individual electrode currents.

The first experimental characterizations of the SiC XBPM, using highly focused (110 nm × 110 nm) 8 keV X-rays, was performed at MAX IV and led to the results described below. The high ratio between the size of the gap and the thickness of the device raised concerns about the possibility of inefficiencies in position measurements in the central region of the gap. Fig. 3 ▸(*a*) shows the measured current of each of the four electrodes on the SiC XBPM, with a nominal active thickness of 250 nm and a 6 µm electrode gap. Fig. 3 ▸(*b*) shows the sum of the measured current from the four electrodes. In regions where no electrode is present, little or no current is measured, indicating substantial loss of CCE. On the other hand, within the electrodes, the measured current shows small, ∼3%, but systematic local fluctuations. These are probably due to the roughness of the membrane, which was estimated to be of the order of 50–100 nm RMS. Given the low membrane thickness, the roughness results in a variation of the thickness and thus in variation of the overall signal. Further measurements will be needed to better clarify this aspect.

**Figure 4:**
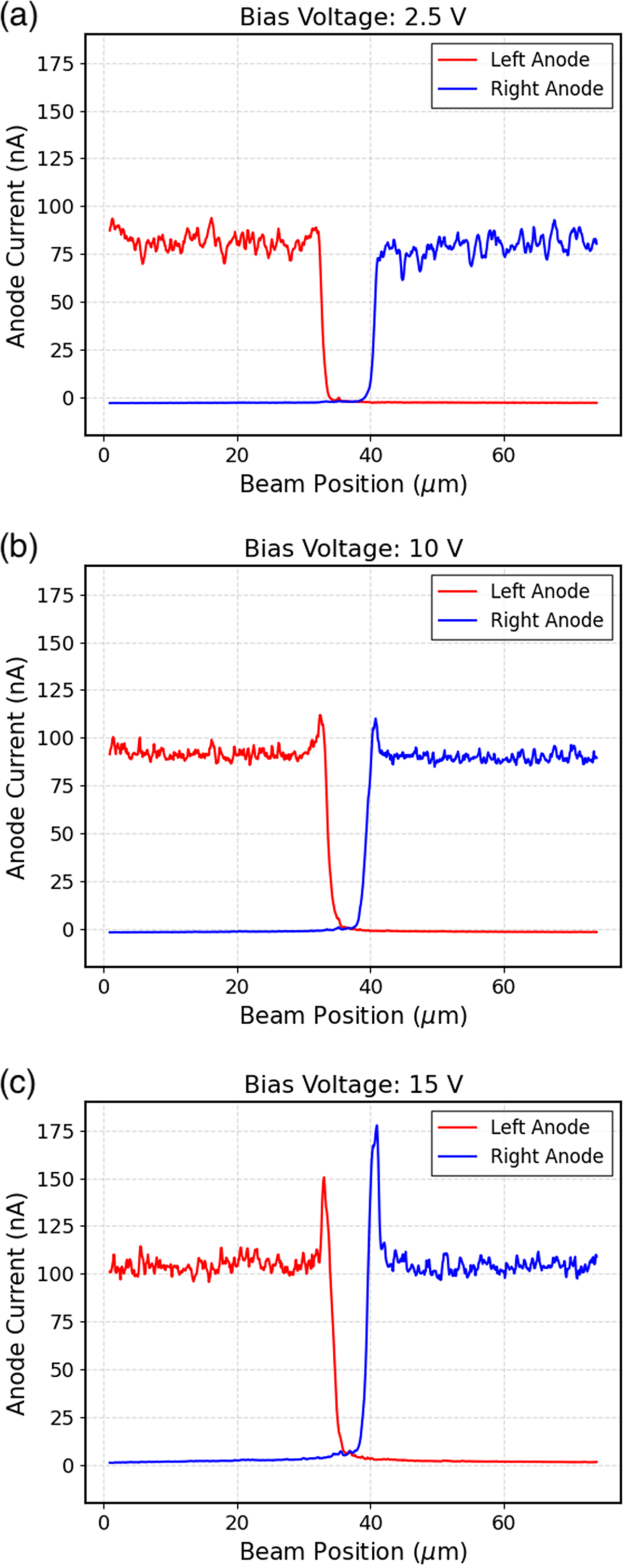
Current measured on two channels (left anode and right anode) as a function of beam position for three different bias voltages (2.5 V, 10 V, and 15 V).

To further understand the response of the sensor as a function of the applied bias, an experimental investigation was conducted by varying the applied bias voltage, thereby modifying the internal electric field. In Fig. 4 ▸, the currents of two adjacent pixels are plotted against the beam position for three different bias voltages. A current peak is observed experimentally at the electrode’s edge, and this peak increases with increasing voltage. Considering the thickness of the sample under examination (250 nm), the bias voltages at which this peak is observed, 10 V and 15 V, correspond to electric field values of 400 kV cm^−1^ and 600 kV cm^−1^, respectively. These results suggest that thin membranes can have a deleterious effect on the CCE of the devices, highlighted by the use of focused X-ray beams.

**Figure 5:**
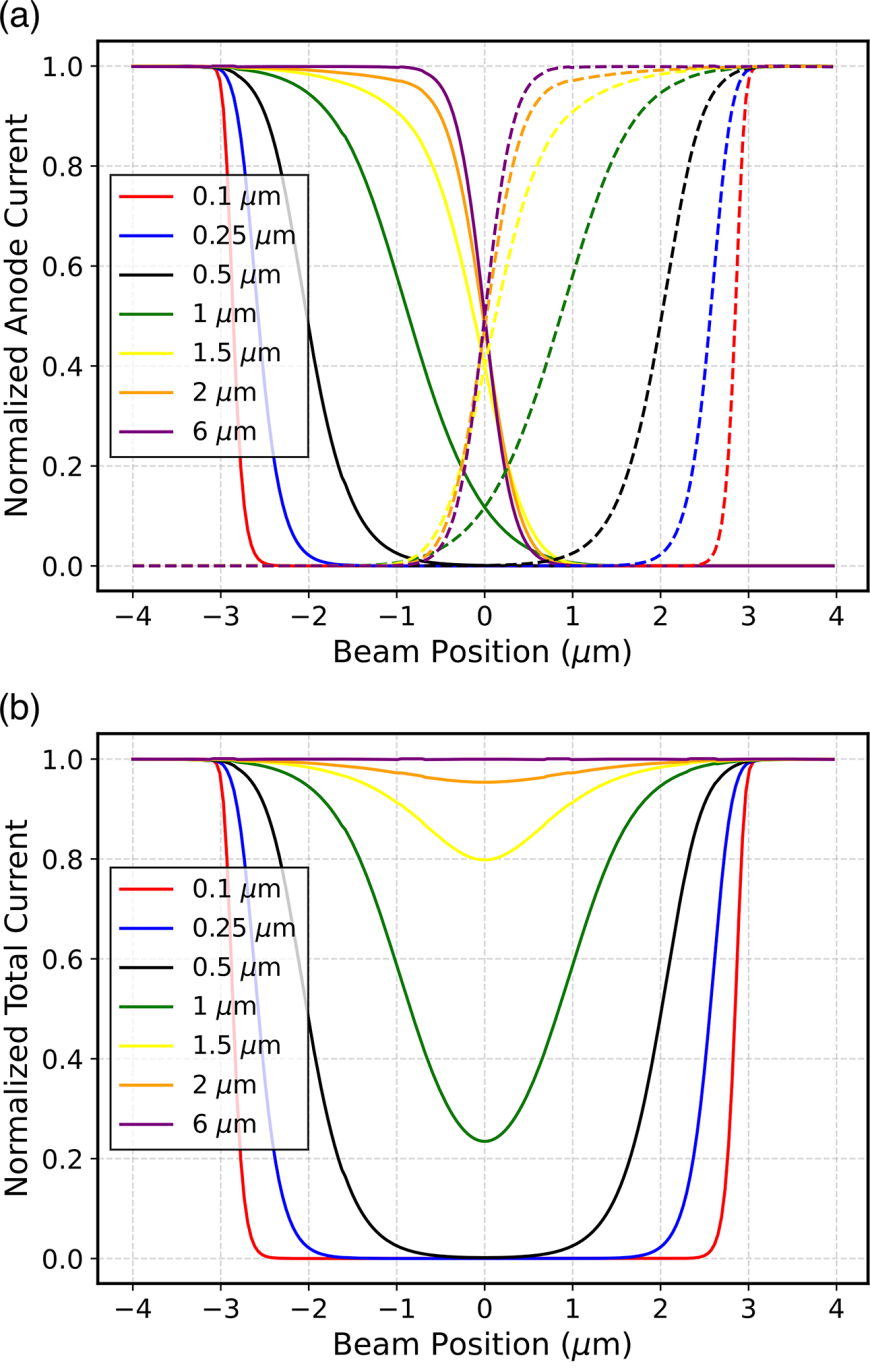
(*a*) Normalized current of left (solid line) and right (dashed line) anodes. (*b*) Total current (sum of the current of the two anodes). The currents are shown as a function of the position of the X-ray beam, for different thicknesses.

In order to consolidate these experimental results, a study on XBPM devices was conducted through simulations using TCAD *Sentaurus* software. Simulations yield the results shown in Fig. 5 ▸.

By scanning a highly collimated beam (FWHM of 100 nm × 100 nm) across two anodes of the sensor, less current is observed in the gap between the anodes, which confirms the observed experimental results (Fig. 5 ▸). This effect was studied as a function of the device thickness. The plot in Fig. 5 ▸(*b*) shows the total current generated in the sensor, calculated as the sum of the two currents of the anodes. In this case, an electric field of 20 kV cm^−1^ was set in the simulation. It can clearly be seen that in the region between −3 µm and +3 µm the beam induced current is reduced by more than 80% for thicknesses of less than 500 nm. This effect diminishes as the thickness increases, until complete charge collection is achieved for thicknesses of the same order of magnitude as the gap. In more detail, from Fig. 5 ▸, it can be seen that the CCE loss is critical only for sensors thinner than 2 µm, confirming that SiC XBPM can operate well in the hard X-ray regimes, as experimentally validated in the literature (Houghton *et al.*, 2023 ▸).

**Figure 6:**
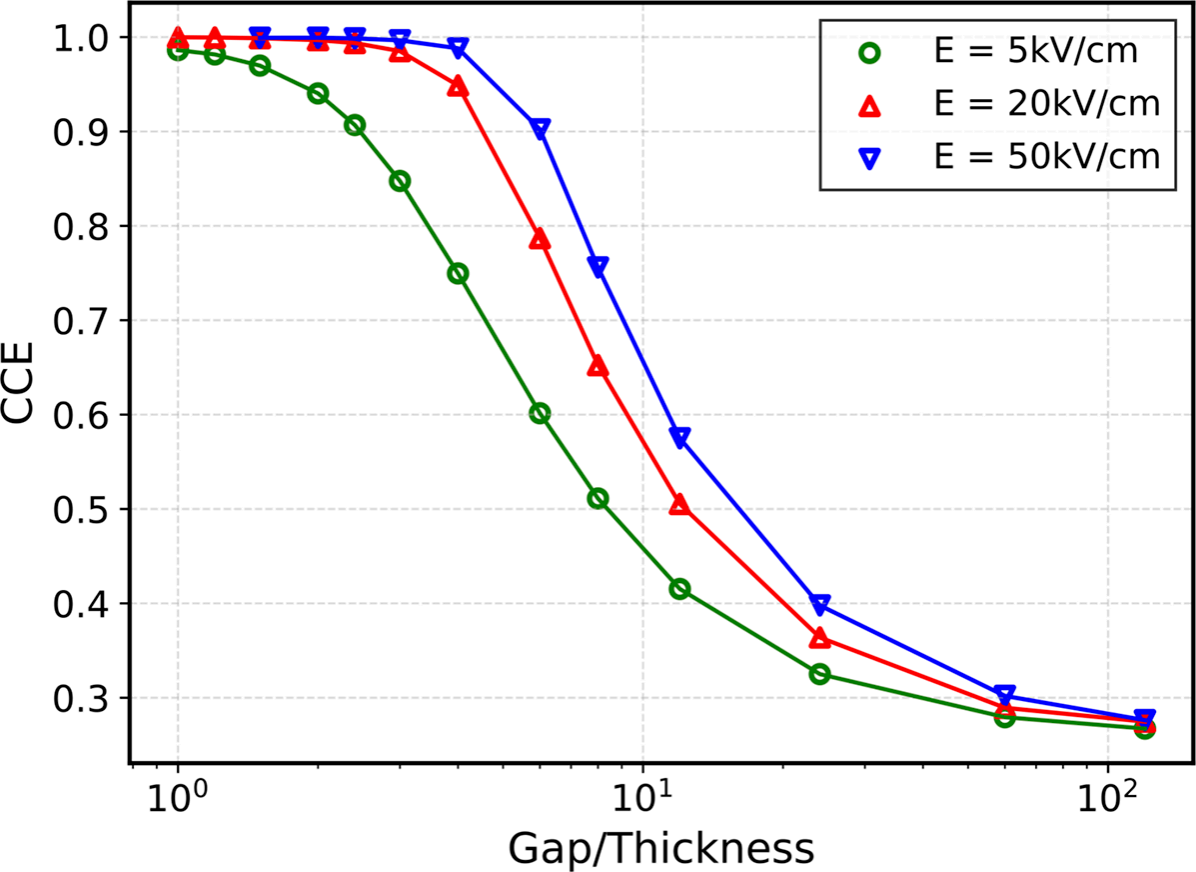
Charge collection efficiency for three electric field values (5 kV cm^−1^, 20 kV cm^−1^, 50 kV cm^−1^) as a function of the ratio gap/thickness.

The CCE was evaluated as the ratio between the integral of the current in the case of a hypothetical complete collection and the integral of the evaluated current. The results are shown in Fig. 6 ▸ where the CCE is represented as a function of the ratio ‘gap/thickness’ for three electric field values (5 kV cm^−1^, 20 kV cm^−1^ and 50 kV cm^−1^). As expected, a higher electric field allows higher collections to be achieved up to a certain limit. Observing Fig. 6 ▸, it can be seen that the CCE in the case of an electric field of 5 kV cm^−1^ exceeds 60% when the ‘gap/thickness’ ratio is approximately less than 4, whereas for an electric field ten times higher (50 kV cm^−1^) at the same ratio it is almost 100%. These results underscore the complexity in monitoring highly collimated beams using sensors with gaps and thicknesses comparable with those previously mentioned. The challenge becomes more pronounced when dealing with very thin sensors. In such cases, precise beam position tracking is only achievable when the FWHM of the beam is significantly greater than the width of the charge collection loss zone. In such a condition, a portion of the signal is captured by the anodes, even when the beam is precisely centered within the gap, so that position sensitivity is still achieved, though at lower lateral sensitivities. However, while the beam position can be monitored, no reliable intensity monitoring is possible due to the loss of signal in the gap region.

**Figure 7:**
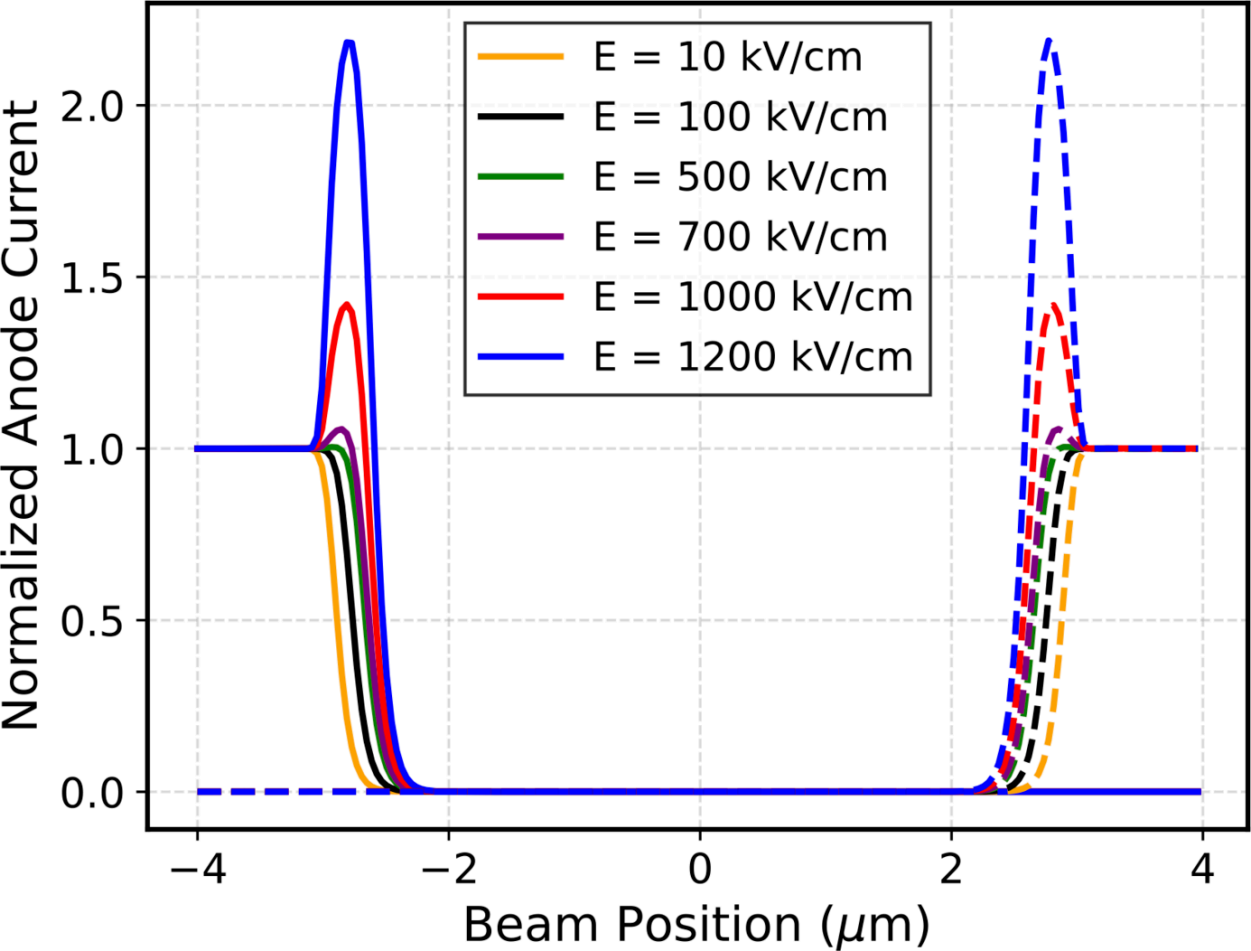
Simulated current of left (solid line) and right (dashed line) anodes as a function of beam position, for different high electric field values (between 10 kV cm^−1^ and 1200 kV cm^−1^).

Similarly to the scans across two electrodes, the signals at very high electric fields have been studied. Fig. 7 ▸ shows the individual anode current measured for a 100 nm thick sensor, normalized with respect to the current measured on the metal anode, under different electric field conditions. It can immediately be seen that an anomalous current rise occurs at the edge between the anode and the central gap when increasing the electric field, consistently with what is observed experimentally. This effect is due to edges of the metals which results in a local electric field increase and consequent internal charge amplification by avalanche effect. In previous simulations, this effect did not arise because lower electric fields were considered.

**Figure 8:**
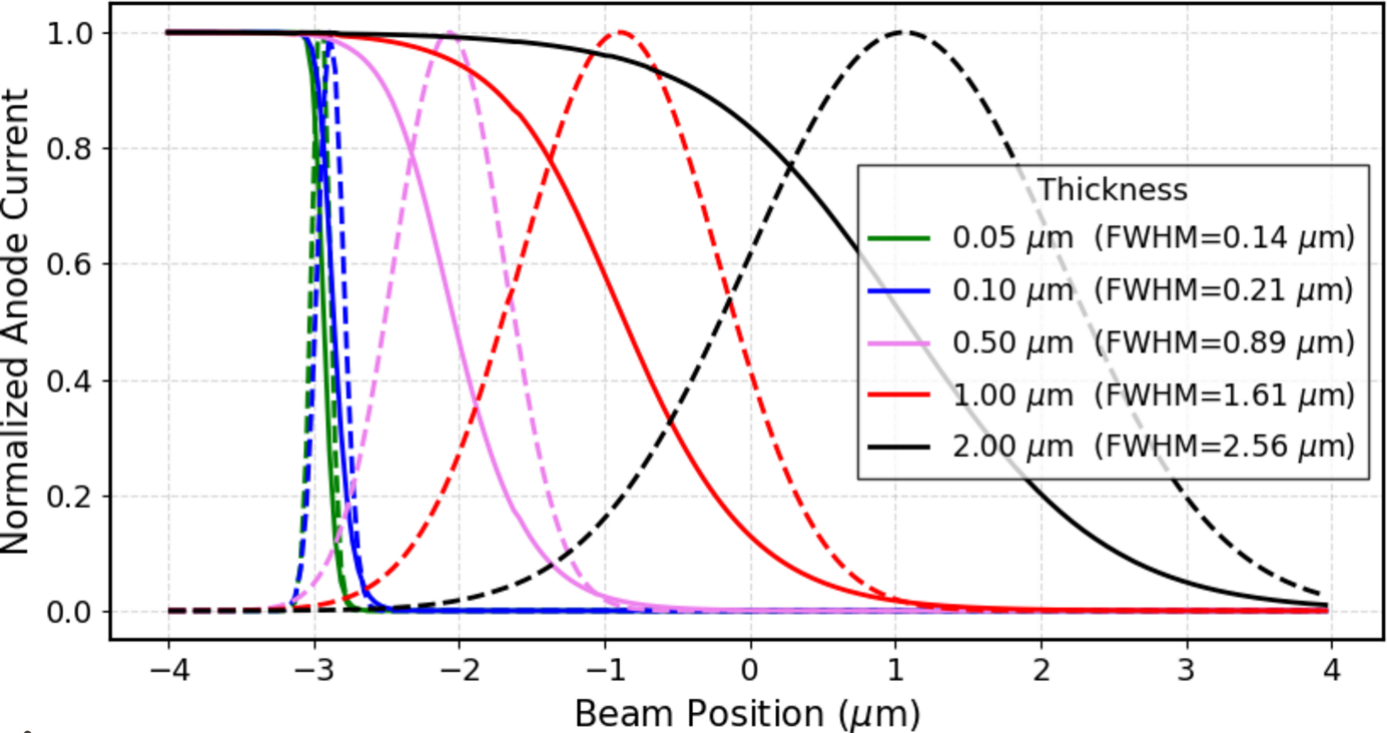
Normalized anode current as a function of beam position measured from one anode (solid lines) for different sensor thickness and their respective double differential (dashed lines).

Despite the results just described, and upon closer examination of Fig. 5 ▸(*a*), it can be observed that, the thinner the sensor, the quicker the current drops when transitioning from an on-anode region to an off-anode region (corresponding to positions −3 µm and +3 µm on the horizontal axes of the plots). This effect is due to the lower carrier diffusion, *i.e.* spreading, of carriers in thinner sensors. This effect can be advantageous for various applications, such as on–off monitoring of the position of even highly collimated beams. The charge spreading width for thin sensors was quantified as the FWHM of the Gaussian obtained by twice differentiating the current as a function of beam position, comparable with an error function. The simulation results are shown in Fig. 8 ▸. The FWHM determined using this method approaches the simulated beam FWHM (100 nm) asymptotically when thinner sensors are employed. However, even with a sensor thickness of 50 nm there is an overestimation of the beam dimension. Based on these results, in order to accurately extract the beam FWHM, it is recommended that the sensor thickness should be at least ten times smaller than the FWHM.

Considering the relevance of the effects observed with the simulations on standard four-quadrant XBPMs, a lower CCE of the device between the electrodes and an important distortion of the electric field in the metal-SiC edge at high voltages, an alternative technology was studied through *Sentaurus* TCAD theoretical simulations: the resistive-XBPMs (rXBPMs).

In these devices, a resistive layer between the active thickness and the readout electrodes is incorporated, thus connecting the 2 × 2 diodes through high-resistance transmission lines. In rXBPMs, the charges generated by incident radiation move and separate, as a current-splitter component, proportionally to the encountered resistivity. The presence of a resistive layer means that the charge is shared between the pads, based on the resistivity towards the four electrodes which, in turn, is related to the charge-to-electrodes distance. This structure makes it possible to increase the fill factor and overcome problems of charge collection loss in the gap between the electrodes, improving spatial resolution with large readout pitch (Tornago *et al.*, 2021 ▸). Devices of this type have already been tested both with silicon, called position sensitive detectors (PSDs) (Mandurrino *et al.*, 2022 ▸; Menzio *et al.*, 2022 ▸; Uesugi *et al.*, 2020 ▸; Cartiglia *et al.*, 2023 ▸), and with diamond (Pomorski *et al.*, 2009 ▸; Çonka Yıldız *et al.*, 2024 ▸). Silicon carbide sensors based on this technology have not been studied yet, and first simulations are given here.

**Figure 9:**
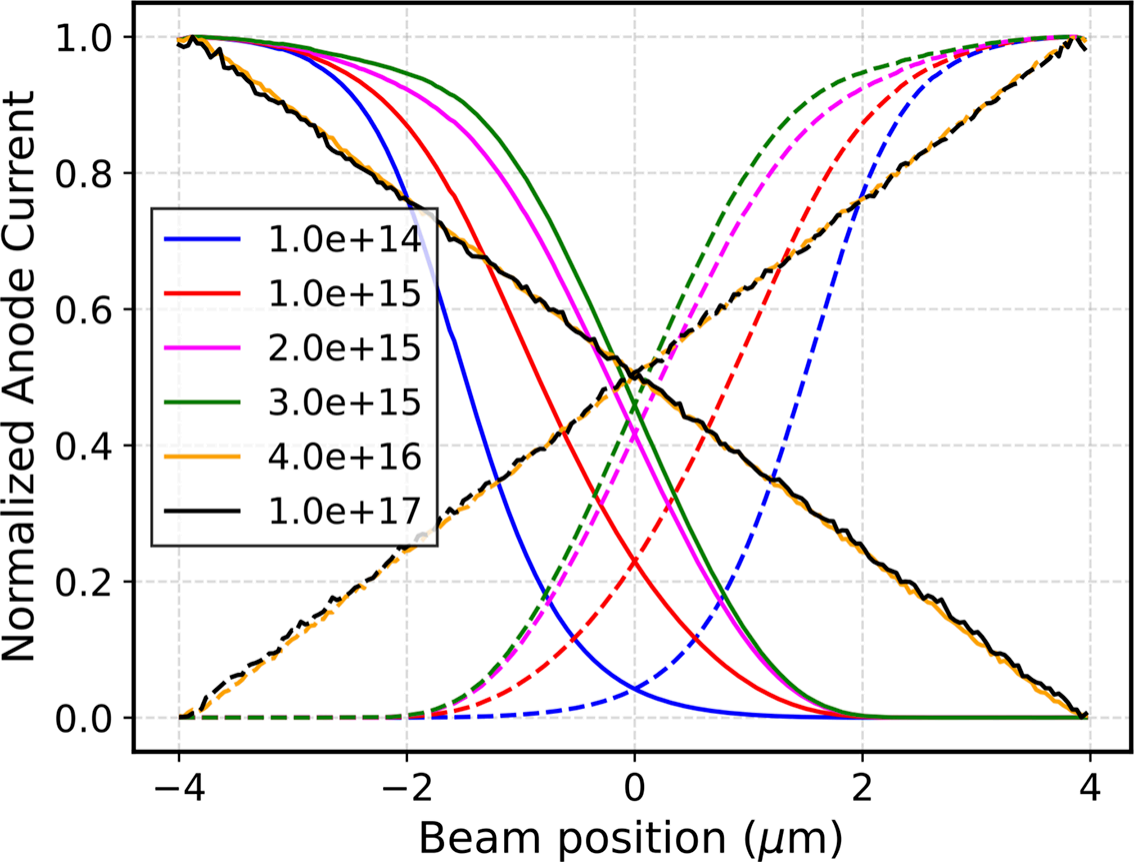
Electrodes simulated current for resistive-XBPM for different values of p^+^ resistive layer doping density (from 10^14^ cm^−3^ to 10^17^ cm^−3^).

To this end, while maintaining the same overall geometry of the ‘standard’ sensors in the TCAD simulations, a thin (100 nm) and continuous p^+^-doped resistive layer was included immediately below the anodes. The anodes, 1 µm in length each, were configured as ohmic contacts. For a device with an active thickness of 1 µm, a study was made varying the doping concentration of the resistive layer, in a range between 10^14^ cm^−3^ and 10^17^ cm^−3^. The results shown in Fig. 9 ▸ highlight that for sufficiently high doping concentrations (> 10^16^ cm^−3^) the trend in the current measured in the individual anodes depends linearly on the distance of the beam from the anode. Additionally, the sum of the current collected at the two anodes maintains a constant value, meaning that no CCE loss is observed at the center of the device.

## 4. Conclusions

SiC sensors are emerging as a promising alternative to current technologies for X-ray position and intensity monitoring. In view of their potential uses for soft and tender X-rays, new ‘four-quadrant’ XBPM devices, developed by SenSiC and featuring very thin Schottky diodes, have been studied and characterized. Their performance was investigated with a 110 nm × 110 nm X-ray beam of 8 keV at the NanoMAX beamline of the MAX IV Laboratory, in Lund, Sweden. The experimental results were compared with the respective simulations using TCAD *Sentaurus Synopsys* software.

Simulations revealed that XBPM devices effectively monitor highly focused X-ray beams (100 nm × 100 nm) only when the gap between electrodes is comparable with the detector thickness. When this condition is not met, as in the case of the tested device, a lower total current is collected across the gap, resulting in a charge collection efficiency loss.

Another observed experimental effect is a current peak at the metal–SiC edge for high applied voltages, attributed to a localized increase in the electric field. Position reconstruction for highly collimated beams is thus challenging and possible only for beams with a FWHM greater than the gap between the electrodes or for sufficiently thick sensors, equal or thicker than 2 µm. These findings indicate that standard four-quadrant XBPMs are unsuitable for soft and tender X-ray, *highly focused* beams, which require SiC membranes thinner than 2 µm. With the aim of addressing this limitation, simulations of silicon carbide resistive-XBPMs, incorporating a resistive layer between the active SiC material and the readout electrodes, were performed. It was observed that, by adding a highly doped resistive layer, a single electrode collects a current linearly proportional to the beam distance. This enables an effective position reconstruction without compromising charge collection. Further simulations will be conducted to determine the optimal doping value for each device geometry. The first devices are currently being manufactured and will soon be explored.
